# Do Zebra Finch Parents Fail to Recognise Their Own Offspring?

**DOI:** 10.1371/journal.pone.0018466

**Published:** 2011-04-13

**Authors:** Hendrik Reers, Alain Jacot, Wolfgang Forstmeier

**Affiliations:** 1 Department of Behavioural Ecology and Evolutionary Genetics, Max Planck Institute for Ornithology, Seewiesen, Germany; 2 Field Station Valais, Swiss Ornithological Institute, Salgesch, Switzerland; 3 IEE-Conservation Biology, University of Bern, Bern, Switzerland; University of Jyväskylä, Finland

## Abstract

Individual recognition systems require the sender to be individually distinctive and the receiver to be able to perceive differences between individuals and react accordingly. Many studies have demonstrated that acoustic signals of almost any species contain individualized information. However, fewer studies have tested experimentally if those signals are used for individual recognition by potential receivers. While laboratory studies using zebra finches have shown that fledglings recognize their parents by their “distance call”, mutual recognition using the same call type has not been demonstrated yet. In a laboratory study with zebra finches, we first quantified between-individual acoustic variation in distance calls of fledglings. In a second step, we tested recognition of fledgling calls by parents using playback experiments. With a discriminant function analysis, we show that individuals are highly distinctive and most measured parameters show very high potential to encode for individuality. The response pattern of zebra finch parents shows that they do react to calls of fledglings, however they do not distinguish between own and unfamiliar offspring, despite individual distinctiveness. This finding is interesting in light of the observation of a high percentage of misdirected feedings in our communal breeding aviaries. Our results demonstrate the importance of adopting a receiver's perspective and suggest that variation in fledgling contact calls might not be used in individual recognition of offspring.

## Introduction

Whenever information is transferred between two individuals, this happens via a signalling system. Signalling systems consist of three parts: the sender, the signal and the receiver. Is the signal used to indicate the identity of the sender to the receiver, for example a young fur seal *Arctocephalus tropicalis* calling for its mother in a nursing colony [1], certain signal properties are needed to ensure reliable recognition by the receiver. The signal must provide between-individual variation, combined with within-individual uniformity, to function as an individual signature [2], [3]. It is well established that between-individual variation in acoustic signals commonly occurs. An extensive body of literature has shown that virtually all acoustic signals of animals across many taxa show between-individual variation [1], [4], [5], [6]. From a receiver's perspective, an animal must be able to perceive these between-individual differences in order to respond accordingly [7].

Acoustic individual recognition is essential in a variety of contexts with repeated social interactions, of which parent-offspring communication has received a lot of attention in recent years [1], [8], [9]. The ability to discriminate calls of single individuals from other conspecifics is especially important in a colony with hundreds or thousands of individuals communicating simultaneously [10], [11]. Parents and their mobile chicks often use calls to reunite after parents have left their offspring alone [12], [13]. Playback experiments have shown that parents and mates perceive acoustic differences between individuals and are able to recognize the sender acoustically [9], [14]. Especially acoustic properties that relate to the time-frequency pattern of a sound (e.g. frequency modulation, frequency range or duration) have been shown to be important for individual distinctiveness and individual recognition [10], [15], [16].

Zebra finches *Taeniopygia guttata*, which are opportunistic breeders with biparental care, nest in loose colonies of up to dozens of pairs, and offspring are fed during an extended post-fledging phase by their parents [17]. Recognition systems are expected to evolve under such breeding conditions, where parents face the risk of potentially confusing their own offspring with other fledglings. The situation, in which recognition of fledglings by its parents is expected to be most important, is after separation when parents and fledglings need to reunite (e.g. after parental feeding trips or predator disturbance). Observations in the wild indicate that fledglings and parents use ‘distance calls’ to reunite [17]. This ability is expected to be very important since the location of a young is a very unreliable indicator of its identity. Especially so in colonially breeding species in which young often change their location and where young are likely to intermingle [1], [10], [18], [19]. Unfortunately, detailed information about the role of parents and their fledglings in the reuniting and recognition process are still missing in the scientific literature. In captivity, it has been shown that young zebra finches respond with distance calls specifically to the distance calls of their parents and mostly ignore calls of other individuals when separated from their parents [20]. However, young occasionally also respond to distance calls of non-parents [17], [20]. In order to avoid feeding unrelated young, parents are expected to be able to recognize their own offspring.

Interestingly however, in communal breeding aviaries of our zebra finch population about 50% of feedings are directed to unrelated offspring [20]. This observation is puzzling considering that Levrero et al. [21] have shown that captive zebra finch parents recognize the begging calls of nestlings one day before fledging. Begging calls are still used once fledged, but only to obtain food in short distance communication, not to reunite after separation [17], [20]. We therefore chose distance calls of fledglings to investigate acoustic individuality and its use in parent-offspring recognition in our captive population. It has been shown that distance calls of adults show individual signatures and are used in mate recognition in which both sexes are able to recognise their partners [15], [16]. However, the ability of parents to use the early distance calls of fledglings to recognise their offspring is unknown and has not yet been experimentally tested. We expect parents to recognize their own fledglings' distance calls for two reasons. First, to make reuniting efficient given the risk that offspring intermingle with conspecific young, and second, to enable parents to invest in their offspring and to avoid feeding of unrelated chicks. In a first step we quantify individual and brood signatures in fledgling distance calls statistically. In a further playback experiment we then test acoustic parental recognition of fledglings with distance calls of their own versus unfamiliar (alien) fledglings.

## Methods

### Ethical Note

The study was approved by the animal care and ethics representative of the Max Planck Institute for Ornithology.

### Study subjects

Fledgling zebra finches used in the present study originated from a captive population held at the Max Planck Institute for Ornithology in Seewiesen, Germany. All fledglings and parents used in this study are from breeding pairs kept in aviaries that held six breeding pairs. The sex of the offspring was determined using molecular methods [22]. Temperature in the rooms was maintained at 24±1°C and relative humidity ranged from 40% to 60%. Rooms were illuminated by full-spectrum fluorescent light (Osram Lumilux T5 FH 28W/860 Daylight) and the light:dark period was 14∶10 h. All birds received a millet seed mixture, egg food (hard-boiled hen's eggs, sprouted millet seed, wheat germ), cuttlefish, grit, water *ad libitum* on a daily basis, and a multivitamin supplement and fresh lettuce once per week. All recognition trials were conducted between May and August 2009. Aviaries were checked twice a day for newly fledged birds. Nestlings were individually marked by numbered alloy bands when eight to twelve days old.

We used distance calls of 84 fledglings recorded in a previous breeding season (2007/2008) to investigate individual distinctiveness using discriminant function analysis (DFA). Calls of these individuals were then used as unfamiliar (alien) stimulus calls for the playback experiment testing parent-offspring recognition. Calls from 64 fledglings from the actual breeding season 2009 were used as ‘own’ stimulus for their social parents in the playback experiment.

### Distinctiveness in fledging distance calls

To investigate acoustic individuality, brood and sex differences in fledgling distance calls, we used 493 calls from 84 fledglings (40 females and 44 males; age: 22.5±2.1 days) originating from 30 broods from the breeding season 2007/2008. For most of the fledglings, no stimulus calls were used during the recording of calls, however for about 10% of fledglings that did not call at all, we used parental calls to stimulate fledgling calling. Fledgling calls were analysed using Sound Analysis Pro software 2.065 [23], a computer program specifically developed for zebra finch vocalizations, using standard settings. We extracted the following acoustic features to characterize the acoustic variability within and between fledglings: (1) call duration (ms), (2) mean amplitude (dB), (3) variance in amplitude modulation, (4) mean frequency (Hz), (5) mean frequency modulation, (6) variance in frequency modulation, (7) mean entropy, (8) variance in entropy, (9) mean pitch (Hz) and (10) mean pitch goodness. These parameters were chosen from a larger pool of parameters because of their usefulness in discriminating between individual zebra finches [20].

### Parent-offspring recognition experiment

We simulated a situation in which a parent lost visual and acoustic contact to its family and where it is expected to react to distance calls of its young. This experimental set-up has proven successful in previous studies of parent-offspring and mate communication in zebra finches [20], [24]. We tested 42 adults from 21 breeding pairs with calls of 64 recently fledged young (mean±SD, 3.0±2.0 fledglings per pair, range: 1–7). The fledglings left the nest at 18.1±1.5 (range: 15–22) days of age and were recorded at 24.5±1.0 (range: 24–28) days of age. The parents were tested 3.1±2.1 (range: 0–8) days after the fledglings were recorded. Both parents were tested with one call from a fledgling of their own (i.e. 128 trials, 64 own fledglings x two parents) against three calls from alien fledglings, which were randomly picked from 84 alien fledglings from the previous breeding season 2007/2008 (N randomly chosen alien fledglings  = 70, number of times used in experiments±SD  = 5.49±2.56). Parents were tested singly in a sound-attenuated recording box (70 cm×50 cm and 50 cm) which was equipped with a small metal wire cage containing a single perch, a microphone (C2, Behringer GmbH, Willich, Germany) approximately 20 cm from the perch and a small loudspeaker (V20, Logitech, Morges, Switzerland) next to the microphone. The microphone was connected to a pre amplifier (SM Pro Audio, Melbourne, Australia) from which we recorded directly through a M-Audio Delta 44 (AVID Technology GmbH, Hallbergmoos, Germany) sound card onto the hard drive of a computer at a sampling rate of 44 kHz and 16 bit amplitude resolution using Audacity 1.3.7 (D Mazzoni, Canada, http://audacity.sourceforge.net/). Audacity was used to play back stimulus calls and to record the parent's response simultaneously. Playback experiments and the previous recording of stimulus calls were done in the same recording box using the same set up.

**Figure 1:**
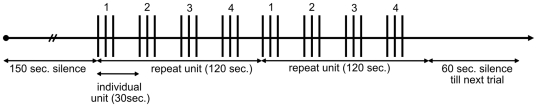
Playback design for fledgling stimulus. Each fledgling stimulus was used with both parents. The order of own and alien stimuli (1, 2, 3, 4) was randomized with the repeat unit.

The playback experiment started with 150 seconds of silence to allow for acclimation of the parent to the recording chamber. For each parent we built a playback that consisted of calls of three different, unfamiliar chicks and one own young. For each of these stimulus birds we build a 30 second ‘individual-unit’ that consisted of the same call starting at 0, 5 and 10 seconds, followed by 20 seconds silence (Figure 1). Four of these individual units, each from a different fledging, were combined into a 120 seconds ‘repeat unit’ that was repeated once and used for both parents in each breeding pair. The order of stimulus individuals (own versus alien) within a repeat unit was randomized. In cases when parents had more than three offspring, we tested no more than three of their fledglings on a given day but continued the experiment the next day. For multiple playbacks on one day, each fledgling's playback was separated by 60 seconds of silence before the playback of the next fledgling started.

The parent's acoustic response was measured as the number of calls within the 5 seconds from the start of each stimulus call. We also measured the latency to call as the time from the start of the stimulus call to the parent's first response call. In cases when parents did not respond to a stimulus, latency was not scored, but the number response call was recorded as zero. Previous studies on zebra finches have shown that the number of calls and the latency to respond are reliable behavioural measures for acoustic recognition in both, adults [16], [21] and young [20]. When comparing locomotor activity response, i.e. adults approaching stimulus calls, with a vocal response, i.e. adults responding to stimulus calls, previous studies in adult zebra finches have shown that the vocal response is a better measure for individual recognition [21], [25].

### Statistical analysis

#### General statistics

All statistical analyses were performed with R 2.8.0 [26] or R 2.10.0 [27].

#### Acoustic individuality

To test whether individuals, broods and sexes can be distinguished statistically by acoustic parameters, we performed three discriminant function analyses (DFA). (1) We used 493 calls from 84 individuals (5.87±0.34 calls/individual±SD) to test for individual distinctiveness. (2) We used averages for each acoustic variable from 43 individuals from 14 broods (i.e. genetic full siblings, no extra-pair young or dumped chicks) with at least two recorded siblings to test for brood differences. (3) We used averages for each acoustic variable from 84 individuals (44 males, 40 females) of each acoustic variable to test acoustic differences between sexes. For the DFAs we used eight call parameters. We excluded variance in frequency modulation because of high inter-correlation (i.e. *r*>0.8) with other parameters (variance in frequency modulation – variance in amplitude modulation, *r* = 0.87; variance in frequency modulation – mean frequency modulation, *r* = 0.85) [28]. In addition, mean amplitude was excluded because variation in amplitude can be due to differences in the direction at which a fledgling is calling towards the microphone. The calls were assigned to individuals, broods or sexes using a cross-validated (leave-one-out method) DFA from the *MASS* package for R [29].

To describe the ratio of intra- to inter-individual variation of each variable, we used the potential for individual identity coding (PIC) [30] and we calculated repeatabilities based on linear mixed-effects models [31]. PIC is a measure of the ratio of inter-individual variation in comparison to intra-individual variation. For each variable we calculated the coefficient of variation (CV) as both, CVi (intra-individual CV) and CVb (between-individual CV) according to the formula:
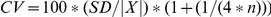
where *SD* is the sample standard deviation, |*X|* is the sample mean and *n* is the sample size [30]. PIC is the ratio of CVb divided by the mean of CVi of all individuals. PIC values above one are considered indicating potential for individual coding because the variation between individuals is larger than within individuals [30]. Repeatabilities were calculated based on linear mixed-effects models fitted by restricted maximum likelihood for all parameters using the *rptR* package [31] for R 2.10.0 [27]. This was done to get a second measure of intra- to inter-individual ratio of variation and to compare PICs and repeatability measurements. Values for PICs and repeatabilities were compared in a correlation analysis. Prior to analysis all acoustic parameters were BoxCox-transformed to approach normality by using the R package *car* [32].

#### Response to playback

To analyze the effects of stimulus calls on a parent's number of response calls, we used generalized linear mixed-effects models (GLMM) from the R package *lme4* [33] with Poisson error-distribution and using sex of the parent (2 levels; i.e. mother, father) and familiarity (2 levels, i.e. own and alien fledgling) as fixed effects. To account for effects of the time the parents had to learn the call of their fledgling or changes in call characteristics between recording and playback, we included the interaction between familiarity and both number of days between fledging and playback (continuous, range: 4–15 days) and number of days between recording and playback (continuous, range: 0–8 days) as fixed factors. As random factors we included parent identity, own fledgling identity and stimulus bird identity. Given that latency cannot be scored when there was no parental response, we only used number of calls as a response measure for GLMMs. The standard model diagnostics of non-normal errors, non-constant error variance and the presence of outliers were performed on each of the final models according to Fox [32].

## Results

### Individual variation in fledgling distance calls

**Figure 2:**
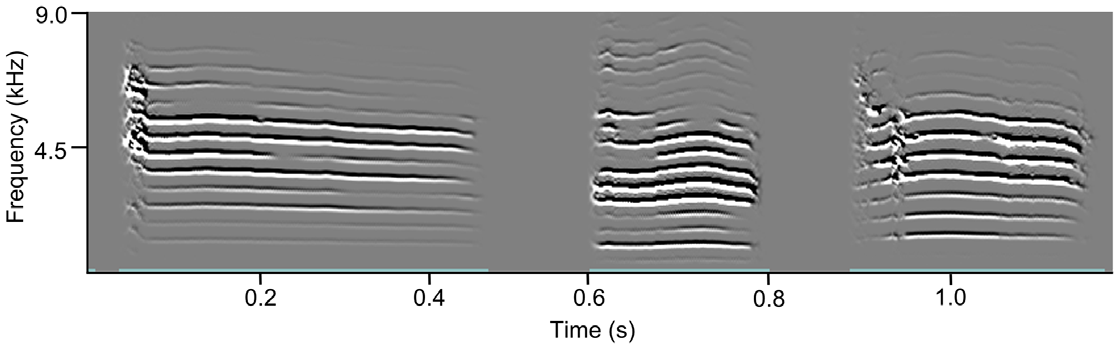
Three representative calls from different individuals showing inter-individual variability of fledgling distance calls.

The cross-validated DFA on 493 calls from 84 fledglings revealed that 70.6% (348 calls) of all calls were assigned to the correct individual. The correct assignment rate was significantly higher than the 1.2% likelihood to be assigned to the correct individual by chance (binomial test: *p*<0.001). The DFA proves that calls provided sufficient individual identity information to be statistically distinguishable (for example calls see Figure 2). In a next step, we investigated which parameters contribute to between-individual variation and provide potential for individuality coding. PIC, i.e. the potential for individual identity coding, showed values well above one for all acoustic parameters. Three out of ten parameters show values above two (Table 1). High repeatabilities and high values for PIC demonstrate that distance calls of fledglings provide a high degree of individuality in all measured call parameters (Table 1). PIC values and repeatabilities were highly correlated (*R^2^* = 0.79, *F_1,8_* = 34.56, *p*<0.001).

**Table 1:** Potential for individual identity coding and repeatabilities for ten acoustic parameters from 493 calls of 84 individuals.

		Potential for individual identity coding				Repeatabilities	
Acoustic parameters		mean±SD (untransformed)	meanCVi	CVb	PIC	R	Confidence interval
Duration (ms)		210.24±62.66	17.56	35.53	2.02	0.83	0.81–0.86
Amplitude	Mean	32.47±4.83	21.60	40.70	1.88	0.85	0.83–0.89
Amplitude modulation (1/ms)	Variance	(5.06±2.56)×10^−3^	24.90	45.86	1.84	0.82	0.80–0.85
Frequency (Hz)	Mean	3933.65±411.20	22.34	32.79	1.47	0.73	0.68–0.77
Frequency modulation	Mean	11.78±6.39	15.89	30.50	1.92	0.81	0.78–0.82
Frequency modulation	Variance	286.62±121.03	24.13	35.82	1.48	0.75	0.68–0.81
Entropy	Mean	−2.51±0.44	14.53	28.05	1.93	0.80	0.77–0.82
Entropy	Variance	0.29±0.15	28.75	35.76	1.24	0.66	0.63–0.71
Pitch (Hz)	Mean	619.68±66.53	13.06	35.37	2.71	0.88	0.85–0.90
Pitch goodness	Mean	829.94±314.53	18.66	40.62	2.18	0.86	0.82–0.86

The DFA on differences between broods of 43 genetic siblings from 14 broods showed a weak effect, 18.6% of fledglings were assigned to the correct brood, which is significantly different from a 7.1% chance of being assigned to the correct brood randomly (binomial test: *p* = 0.01). The DFA on sex differences of fledglings showed that 53.6% of 84 individuals were assigned to the correct sex, which is statistically not different from a by chance correct assignment rate of 50.0% (binomial test: *p* = 0.59).

### Vocal recognition of nestlings – a playback experiment

**Figure 3:**
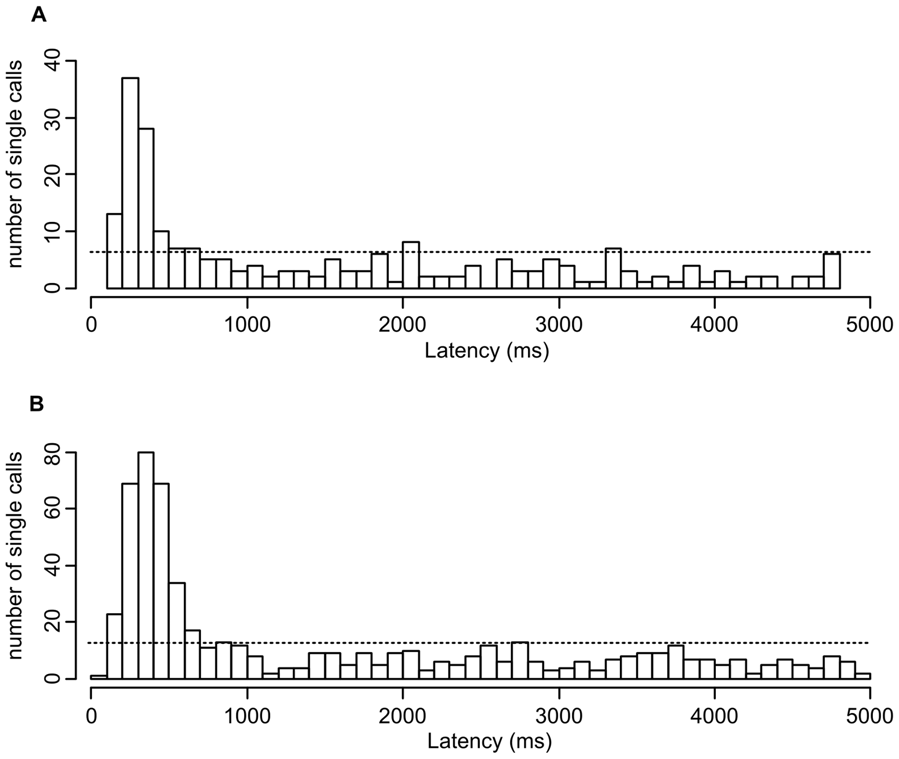
Histogram plots of single response calls in relation to latency of response to (A) own and (B) alien fledglings. Most responses are given shortly after the stimulus, which shows that calls are emitted in response to the stimulus. The dashed line indicates average response frequency of random response latency. Response frequencies for alien fledglings are three times higher, caused by a 1∶3 ratio of own versus alien stimuli per adult.

**Figure 4:**
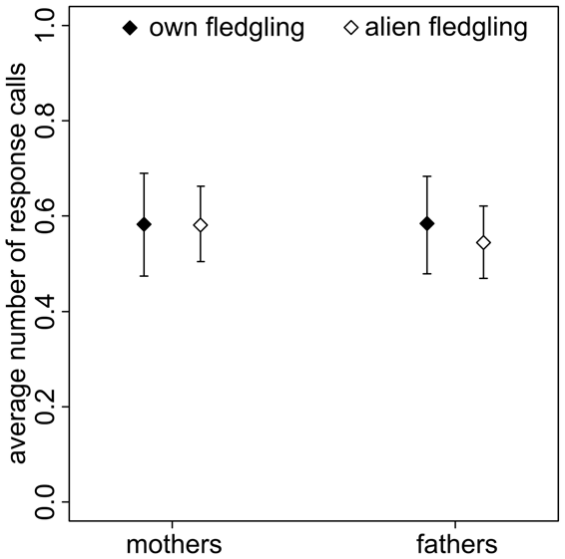
Average number of response calls (± standard errors) for mothers and fathers to own and alien fledgling calls.

To test the ability of parents to recognize their own offspring acoustically, we tested 42 parents (21 breeding pairs) with calls of 64 fledglings (1–7 per brood). Three adults did not respond to any stimulus. Most responses to playbacks of either own or alien fledglings were single calls (Table 2). The response pattern for responses with only one call showed that calls were emitted specifically in response to the stimulus (median (*Q1_25%_/Q3_75%_*): *own*: 900 ms (330 ms/2671 ms); *alien*: 719 ms (366 ms/2664 ms); Figure 3) and not in a random pattern, where the median latency would be around 2500 ms. The latency of an adult to respond to a stimulus was related to the number of response calls (Spearman rank correlation, r_s_ = −0.26, *p*<0.0001, *N* = 1205 responses). The number of response calls in response to the stimulus calls revealed that parents did not respond differently to own or alien fledgling (GLMM: *b*±*SE*  = −0.03±0.06, *t* = −0.60, *p* = 0.55, *N* = 42, Figure 4) nor did the sex of the parents affect the overall responsiveness (GLMM: *b*±*SE*  = 0.56±0.42, *t* = 1.34, *p* = 0.18, *N* = 42, Figure 4). The response pattern of adults was not related to the number of days the fledgling had left the nest (interaction familiarity x number of days fledged: GLMM: *b*±*SE*  = 0.02±0.03, *t* = 0.53, *p* = 0.59, *N* = 42) nor was it related to the number of days between the recording and the playback (interaction familiarity x number of days between recording and playback: GLMM: *b*±*SE*  = −0.03±0.04, *t* = −0.78, *p* = 0.43, *N* = 42).

**Table 2:** Counts of how often adults responded with a certain number of calls to stimuli from own or alien fledglings and the according percentage of the overall response.

	Number of response calls per stimulus							
	0	1	2	3	4	5	6	7
own	456	224	63	19	5	1	0	0
fledglings	59.4%	29.2%	8.2%	2.5%	0.7%	0.1%	0.0%	0.0%
alien	1411	588	212	60	21	7	4	1
fledgling	61.2%	25.5%	9.2%	2.6%	0.9%	0.3%	0.2%	0.0%

Responses for alien are approximately three times higher than own, due to a 1∶3 ratio of own versus alien stimuli per adult.

## Discussion

The results of this study show that fledgling distance calls are highly individually distinct and can be discriminated statistically, based on their acoustic properties. Further, we demonstrate that adult zebra finches do call in response to playback of fledgling distance calls, however, they do not respond differently to the calls of their young compared to alien young.

DFA failed to discriminate the sex of fledglings using acoustic parameters from distance calls. This suggests that distance calls presumably contain very little to no information about the sex of a fledgling, at least when considering the acoustic parameters measured in the present study. Acoustic differences between broods were rather weak; the DFA on brood differences assigned nestlings to broods correctly only for a small number of nestlings. This suggests that parents would most likely be unable to distinguish between their own and alien fledglings based on a common signature that all fledglings from one brood share. Consequently, parents would need to recognize their fledglings individually to discriminate them from foreign offspring, instead of using a brood signature for all their offspring.

Levréro et al. [21] have shown that begging calls of young zebra finches contain individualized information one day before the nestlings leave their nest. In contrast to begging calls used in the study by Levréro et al. [21], we have used distance calls of offspring shortly after fledgling. Those calls are more similar to the adult distance calls and consist of a single call compared to the begging call, which contains a train of calls and is only used in begging situations [17], [21]. The DFA on distance calls of young fledglings demonstrates that calls provide individual information sufficient to discriminate statistically between fledglings. The individuality in fledgling calls, as measured by the DFA, is more similar to distance calls of adult zebra finch females (i.e. 72%, *N_females_* = 94, using 10 acoustic parameters), but less than in distance calls of adult males (i.e. 95%, *N_males_* = 100, using 10 acoustic parameters) [20]. PIC values show that every measured parameter shows very high levels of individuality.

In the playback experiment, parents responded specifically to stimulus calls and not in a random manner (Figure 3). However, despite the high degree of acoustic individuality in distance calls of fledglings, parents did not respond specifically to their own offspring. Do parents lack the perceptual abilities to distinguish between their own and foreign fledglings based on distance calls? Although some studies do not find parental recognition of chick vocalizations [34], [35], [36], this seems unlikely for the zebra finch. Previous studies have shown that adult zebra finches are able to recognize mates using distance calls [15], [16]. Zebra finches even showed the ability to discriminate between individual humans based on the speakers voices [37]. Fledgling distance calls provide similar acoustic individuality to adult distance calls (see above), parents should therefore be able to perceive acoustic differences between fledglings.

It is possible that the lack of specificity in parental response is due to a lack of motivation in relation to the experimental set-up. Parents may not respond stronger in order to minimize the risk of the chicks getting into a dangerous situation. However, previous studies on parent-offspring communication and on the genetic basis of zebra finch vocalizations using the identical experimental setup have shown that zebra finches reacted in a more or less natural way [20], [24]. It remains possible that chick recognition shortly after fledging is primarily based on variation in begging. After fledging, recognition of distance calls in zebra finches may not be mutual but one-sided and parent-offspring recognition may follow a two-step process. In a first step, fledglings could recognize distance calls of their parents, respond with their own distance call [20] and move in the direction of their calling parents. Once the fledgling is reunited with its parents, parents may use the fledglings obligatory begging display, emitted to solicit food, to acoustically recognize their fledgling and avoid costly false feeding [21]. This scenario conflicts with the high rate of false feedings in our breeding aviaries [20], which, however, might be a consequence of the close proximity of breeding pairs within the aviary situation. In the wild, Zann [17] described that parents and fledglings use distance calls to reunite, but did not give details about the exact sequence of calling and approach behaviour. Future studies in the wild or in larger aviaries allowing spatial separation might be able to clarify the importance of begging and distance calls in parent-offspring recognition and parental feeding patterns.

Although we were able to demonstrate that fledgling distance calls show potential for individual recognition, we cannot conclude that distance calls are actually used by parents to recognize their offspring. This highlights the importance of adopting a signaller's and a receiver's perspective in a signalling system. Just demonstrating individuality in a signal does not imply that this information is used for individual recognition by the receiver. This study also points out, that complex social interaction might be altered by laboratory conditions and therefore might only be fully understandable in a more natural context. Further field and laboratory studies are clearly needed to understand the complexity of vocalizations and their functions in different social contexts.
